# Supplementary material for: Maternal Exercise Rescues Fetal Akinesia‐Impaired Joint and Bone Development
Source: FASEB J. 2025 Dec 13;39(24):e71341. doi: 10.1096/fj.202503192R (PMC12701528; doi:10.1096/fj.202503192R)
Supplement: Supplementary file 1 — Figure S1: Wheel acclimation and maternal exercise regimen. (A) Schematic representation of maternal exercise modalities. Female mice were acclimated to running wheels with ad libitum access for 2 weeks prior to timed pregnancies. After timed pregnancies, pregnant dams had no wheel access until embryonic day 13.5 (E13.5). At E13.5, pregnant dams were assigned to one of two exercise modalities: (1) Ad libitum wheel access until harvest at E17.5 or (2) supervised wheel running daily until harvest at E17.5. Each exercise modality included Sham mice, which had the same wheel exposure, but wheels were locked so that the pregnant dams could not use them to exercise. Embryos were harvested at E17.5 for downstream analyses. (B) Daily running distance throughout the 2‐week acclimation period. (C) Daily running distance for pregnant dams using the ad libitum exercise modality. (D) Daily running distance for pregnant dams using the supervised running exercise modality. Each line represents one pregnant dam. (E) Schematic and legend for pregnant C57BL/6J dams used in this study. Each pregnant dam is noted by a different shade of blue for Sham animals and red for exercise animals. (F) Maternal age, (G) maternal mass at E17.5, and (H) litter size. Figure S2: Optical projection tomography (OPT) imaging of fetal forelimbs. (A) OPT apparatus with a high‐solution camera, a rotating stage, and a magnetic sample holder marked by yellow arrows. (B) Example process for how a series of 400 brightfield images captured at 360° was reconstructed and converted into a 3D model. Figure S3: Analysis of covariance (ANCOVA) for effect of maternal exercise on humerus bone length, accounting for variation in fetal weight. (A) Single linear regression fitting humerus length vs. fetal weight for Sham and exercise fetuses. (B) Separate linear regressions fitting humerus length vs. fetal weight for Sham and exercise fetuses. Bands indicate 95% confidence interval for each curve. Goodness of fit (R2) and [file FSB2-39-e71341-s001.pdf]

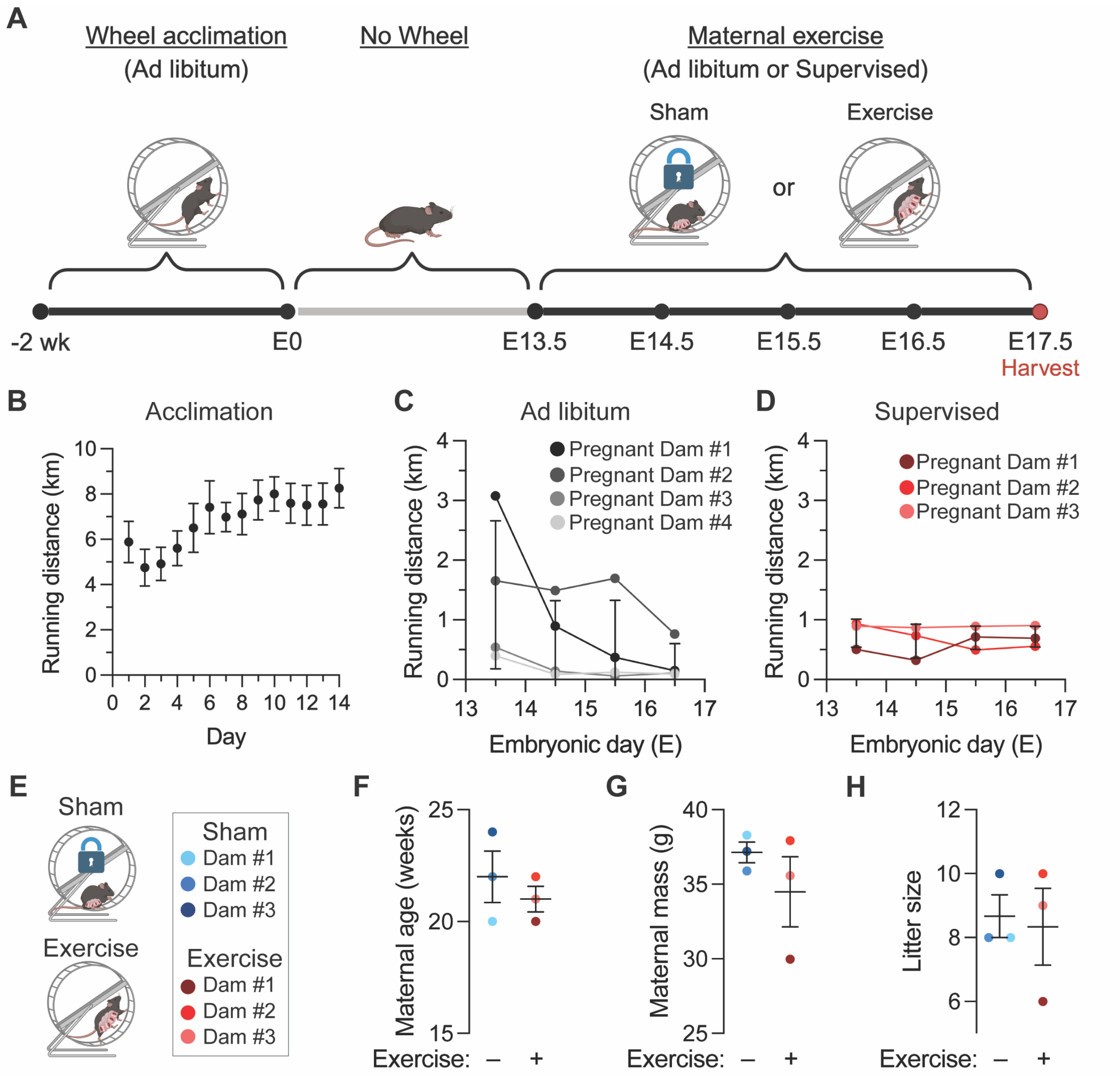

Supplemental Figure 1. Wheel acclimation and maternal exercise regimen. (A) Schematic representation of maternal exercise modalities. Female mice were acclimated to running wheels with *ad libitum* access for two weeks prior to timed pregnancies. After timed pregnancies, pregnant dams had no wheel access until embryonic day 13.5 (E13.5). At E13.5, pregnant dams were assigned to one of two exercise modalities: (1) *Ad libitum* wheel access until harvest at E17.5 or (2) Supervised wheel running daily until harvest at E17.5. Each exercise modality included Sham mice, which had the same wheel exposure, but wheels were locked so that the pregnant dams could not use them to exercise. Embryos were harvested at E17.5 for downstream analyses. **(B)** Daily running distance throughout the two-week acclimation period. **(C)** Daily running distance for pregnant dams using the *Ad libitum* exercise modality. **(D)** Daily running distance for pregnant dams using the Supervised running exercise modality. Each line represents one pregnant dam. **(E)** Schematic and legend for pregnant C57BL/6J dams used in this study. Each pregnant dam is noted by a different shade of blue for Sham animals and red for Exercise animals. **(F)** Maternal age, **(G)** maternal mass at E17.5, and **(H)** litter size.

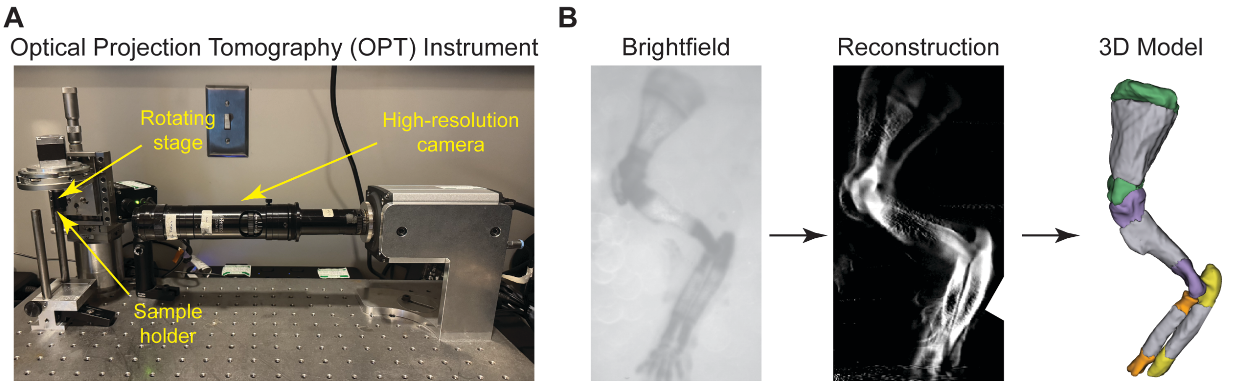

Supplemental Figure 2. Optical projection tomography (OPT) imaging of fetal forelimbs. (A) OPT apparatus with high-solution camera, rotating stage, and magnetic sample holder marked by yellow arrows. **(B)** Example process for how a series of 400 brightfield images captured at 360° was reconstructed and converted into a 3D model.

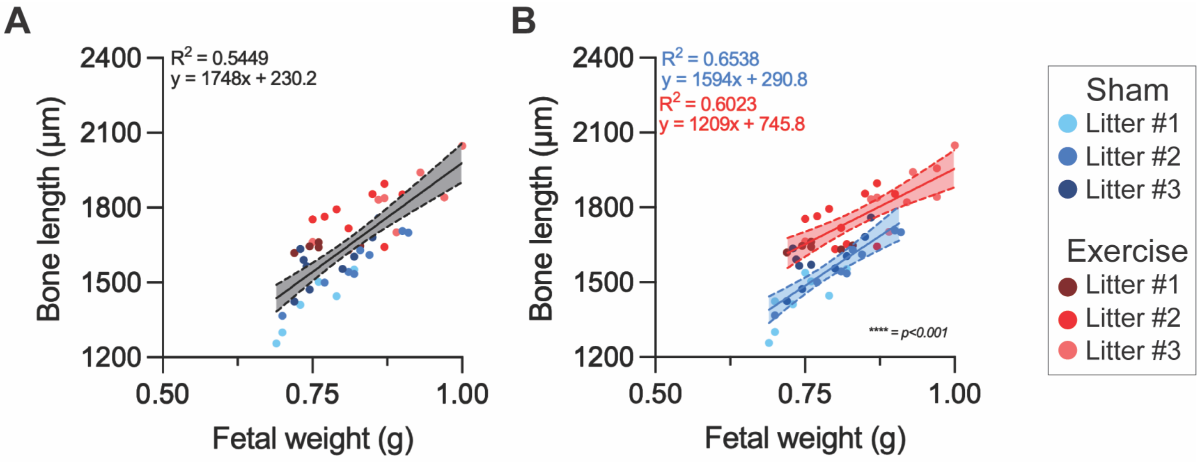

Supplemental Figure 3. Analysis of covariance (ANCOVA) for effect of maternal exercise on humerus bone length, accounting for variation in fetal weight. (A) Single linear regression fitting humerus length vs. fetal weight for Sham and Exercise fetuses. **(B)** Separate linear regressions fitting humerus length vs. fetal weight for Sham and Exercise fetuses. Bands indicate 95% confidence interval for each curve. Goodness of fit (R^2) and linear equation are reported for each linear regression.
